# Associations between Participant Characteristics and Participant Feedback about an Unsupervised Online Cognitive Assessment in a Research Registry

**DOI:** 10.14283/jpad.2023.40

**Published:** 2023-04-19

**Authors:** Miriam T. Ashford, J. Eichenbaum, C. Jin, J. Neuhaus, A. Aaronson, A. Ulbricht, M. R. Camacho, J. Fockler, D. Flenniken, D. Truran, R. S. Mackin, P. Maruff, M. W. Weiner, R. L. Nosheny

**Affiliations:** 1 VA Advanced Imaging Research Center, San Francisco Veteran’s Administration Medical Center, San Francisco, CA USA; 2 Department of Veterans Affairs Medical Center, Northern California Institute for Research and Education (NCIRE), 4150 Clement Street, San Francisco, CA 94121 USA; 3 San Francisco Department of Radiology and Biomedical Imaging, University of California, San Francisco, CA USA; 4 Department of Epidemiology and Biostatistics, University of California San Francisco, San Francisco, CA USA; 5 CogState, Melbourne, VIC Australia; 6 Department of Psychiatry, University of California San Francisco, San Francisco, CA USA; 7 Department of Neurology, University of California San Francisco, San Francisco, CA USA; 8 Department of Medicine, University of California San Francisco, San Francisco, CA USA

**Keywords:** Brain health registry, feedback, race, education, Cogstate Brief Battery

## Abstract

**Background:**

This study aims to understand whether and how participant characteristics (age, gender, education, ethnocultural identity) are related to their feedback about taking a remote, unsupervised, online cognitive assessment.

**Methods:**

The Brain Health Registry is a public online registry which includes cognitive assessments. Multivariable ordinal regressions assessed associations between participant characteristics and feedback responses of older (55+) participants (N=11,553) regarding their Cogstate Brief Battery assessment experience.

**Results:**

Higher age, secondary education or less, Latino identity, and female gender were associated with a poorer assessment experience; higher age and a non-White identity were associated with experiencing the assessment instructions as less clear; and higher age, non-White identity, and secondary education or less were associated with rating additional human support with the assessment as more useful.

**Discussion:**

Our findings highlight the importance of improving the design and instructions of unsupervised, remote, online cognitive assessments to better suit the needs of diverse communities.

## Introduction

As access, literacy, acceptance, and usage of digital technology and the internet increases among older adults, including those from diverse ethnocultural and socioeconomic communities (1, 2), unsupervised online cognitive assessments represent a promising approach to efficiently evaluate cognition in age-related brain disease, such as mild cognitive impairment (MCI) and Alzheimer’ disease (AD).

By allowing individuals to interact with cognitive assessments at the time and place of their choosing, unsupervised online cognitive assessments could allow greater access to geographically and ethnoculturally diverse communities, allow researchers to access larger study samples with lower burden (e.g., time) and costs on their participants, provide opportunities for repeated assessments at both short and long re-test intervals, and may thereby provide older adults with, or at risk of AD greater access to clinical care, clinical research, and potentially clinical trials (3). The importance of remote assessments has also been highlighted by the strategies used to deliver health care during the COVID-19 pandemic (4). To understand the limits of unsupervised online cognitive assessments, including the extent to which they provide data that are equivalent to in-clinic supervised assessment, data of in-clinic supervised assessments can be compared to data from online unsupervised assessments. Another approach is to examine relationships between performance on unsupervised cognitive assessments and AD-related biomarkers to obtain estimates of criterion validity (3). Numerous challenges to the validity of data collected from online unsupervised assessments remain. For example, data quality can be impacted by external events such as the environment in which the assessment is taken. In addition, assessment comprehension, motivation, and completion could be reduced by the absence of an assessor (5, 6). Further, cognitive assessments used in remote contexts are often developed and validated in highly-educated non-Latino White communities (7, 8). This bias is likely to limit the validity and potential generalizability of assessment findings when they are applied in diverse ethnocultural and socioeconomic communities that do not have these characteristics. While there is promising evidence for assessment adherence in studies conducted in highly-educated non-Latino White individuals (9), retention in longitudinal assessment studies of diverse participants is especially challenging (10), which can also impact the generalizability of the collected data.

Recently, several online AD-related research and recruitment registries have been established to efficiently recruit and assess cognition and health in older adults (11–14). Although they require access to technology and the internet and some digital literacy, online assessments in registries might be able to support assessment completion and retention within populations that are not commonly included in clinical research, for example, through culturally adapting assessments’ design and instructions, including availability in multiple languages.

The Brain Health Registry (BHR) is a voluntary online research and recruitment registry which supports remote, unsupervised, online cognitive assessment (14). Analyses of BHR data and comparison with data gathered in supervised in-clinic settings provide evidence for the feasibility and validity of unsupervised cognitive assessments (14, 15). Despite some on-going efforts, the BHR has not been effective at engaging and retaining older adults from diverse ethnocultural and socioeconomic communities in the United States (14, 16, 17). Acceptability and usability in remote cognitive assessment have previously been evaluated by examining completion data and test performance errors in registries of cognitive aging, but little information about views from participants about their experience of taking online, remote, unsupervised cognitive assessments exist (9, 18–21). In this context, insights based on the feedback of participant from diverse ethnocultural and socioeconomic communities could help guide improvements in the design and instructions of online cognitive assessment. This may ultimately improve understanding, adherence, and completion both at baseline and longitudinally of participants from diverse ethnocultural and socioeconomic communities.

The aim of this study was therefore to evaluate BHR participants’ self-reported experience of taking an unsupervised, online cognitive assessment. More specifically, this study aimed to understand whether and how the characteristics of BHR participants aged over 55 years, such as age, gender, education, ethnocultural identity, are related to their feedback responses about taking an unsupervised, online cognitive assessment in BHR. This included a rating of the assessment experience (poor vs excellent), clarity of assessment instructions, and usefulness of additional personal help with assessment instructions. Based on previous BHR assessment engagement analysis results[16], we hypothesized that increasing age, self-identifying as non-White, and having a lower level of educational attainment are associated with poorer assessment experience.

## Methods

### Study setting and samples

The Brain Health Registry (BHR) is a public, online, voluntary recruitment and research registry for the assessment, longitudinal monitoring, and referral of participants to other online and in-clinic studies of aging (14). BHR was developed by University of California, San Francisco researchers in 2014 and is approved by UCSF Institutional Review Board. Since its inception, over 90,000 participants have enrolled. Participants must be aged 18 years or older and complete an electronic informed consent before being invited to complete a series of unsupervised online self-report questionnaires (e.g., sociodemographic information, health-related questions, medical history, depression, memory complaints, family history of AD) and different cognitive assessments every six months. Participants are not compensated for completion of tasks. For more information about BHR see Weiner et al. 2018 (14). This analysis included participants who answered optional rating-scale questions regarding their cognitive assessment experience and were aged 55 or older (55+, N=11,553).

### Measures

#### Unsupervised online cognitive assessment feedback metrics

Participants enrolled in BHR complete unsupervised online cognitive assessments every six months. One of the BHR cognitive assessments is conducted using the Cogstate Brief Battery (CBB). The CBB is a computerized cognitive assessment battery which consists of four subtests: (i) Detection test (information-processing speed, attention, motor speed); (ii) Identification test (visual attention); (iii) One-Card Learning test (visual learning, memory); (iv) One-Back test (working memory). The CBB has been validated under supervised and unsupervised conditions in various populations, including aging and ADRD studies, different language groups, tribal indigenous groups, and in developing countries (20, 22–25). After completing the CBB, BHR participants are invited to answer three optional feedback questions (hereafter referred to as “post-CBB feedback questions”) about: (1) their test taking experience (“How would you rate your experience taking this test?”) rated on 5-point scale (1=Poor, 2=Fair, 3=Good, 4=Very Good, 5=Excellent); (2) clarity of test instructions (“Were the instructions clear?”) rated on a 4-point (1=Not Very Clear, 2=Somewhat Clear, 3=Very Clear); and (3) usefulness of additional personal help with test instruction (“Do you think it would have been helpful for someone to explain this test to you and answer your questions before starting?”) rated on a 4-point scale (1=Not Useful, 2=Not Very Useful, 3=Somewhat Useful, 4=Very Useful). These questions were internally developed in collaboration with a marketing partner. This analysis included the first instance at which BHR participants answered these three feedback questions after completing the CBB assessment. We also retrieved information about which version of CBB (Flash vs HTML5) participants completed.

#### Participant characteristics metrics

Enrolled BHR participants complete a variety of online self-report questionnaires. For this analysis, we included data from the following participant characteristics: gender (male, female, other, prefer not to say), age (continuous), race (Asian, African American/Black, Caucasian/White, Native American, Pacific Islander, other, decline to state), ethnicity (Latino, non-Latino, declined to state), and educational attainment (categorical). The categorical variable educational attainment was converted into a 3-level variable called levels of educational attainment (secondary or less: grammar school, high school; post-secondary: some college, two-year degree, four-year degree; post-graduate: Master’s degree, doctoral degree; professional degree). We also created an ethnocultural identity variable (Latino, non-Latino Black, non-Latino Asian, non-Latino White, other non-Latino).

### Statistical analyses

The objective of this statistical analysis was to determine associations between sociodemographic variables (age, gender, levels of length of education, ethnocultural identity) and the responses to the three post-CBB feedback questions. Descriptive statistics were calculated including frequencies and percentages for categorical data and mean and standard deviation (SD) for continuous data to assess participant characteristics and answers to the feedback questions. For assessing the associations, we employed a series of multivariable ordinal logistic regression models. We fit separate ordinal models to the three ordinal feedback responses and included all of the sociodemographic variables as the predictors. In addition, CBB version (Flash vs HTML) was added as a covariate, since Flash was being phased out by operating systems while CBB on BHR still ran on Flash. During this time, we experienced an influx of participants contacting us with CBB issues, and we included CBB version to account for this. We report odds ratios (OR) and 95% confidence intervals (CI) for the models. SAS 9.4 (SAS Institute, Cary NC) was used for all statistical analyses.

## Results

### Sample characteristics

Of all BHR participants enrolled at the time of the study (N=87,825), 56,756 participants were aged 55+. Of those age 55+, 11,553 (20.4%) provided post-CBB feedback. See Table 1 for participant characteristics. The mean age of those who provided post-CBB feedback was 66.3 (SD=7.08), 71.7% identified as female, the mean years of education was 16.2 (SD=2.39) and 85.8% identified as non-Latino White.

**Table 1:** Descriptive statistics of participant characteristics and feedback ratings for BHR participants ≥55 years

	**BHR participants aged 55+ N=56,756**	**BHR participants aged 55+ who completed the post-CBB feedback questions N=11,553**
Age, Mean (M) ± Standard Deviation (SD) (range)	65.8 ± 7.4 (55–90)	66.3 ± 7.08 (55–90)
Education in years, M ± SD (range)	15.8 ± 2.54 (6–20)	16.2 ± 2.39 (6–20)
Levels of educational attainment, n(%)		
Secondary or less	4236 (7.5%)	572 (5.0%)
Post-secondary	31857 (56.1%)	6206 (53.7%)
Post-graduate	19596 (34.5%)	4760 (41.2%)
missing	1067 (1.9%)	15 (0.1%)
Female, n(%)	41042 (72.3%)	8270 (71.6%)
Ethnocultural identity, n(%)		
Latino	6506 (11.5%)	737 (6.4%)
Non-Latino Black	1715 (3.0%)	165 (1.4%)
Non-Latino Asian	959 (1.7%)	192 (1.7%)
Non-Latino White	42055 (74.1%)	9911 (85.8%)
Non-Latino Other	1923 (3.4%)	360 (3.1%)
Not available	3598 (6.3%)	188 (1.6%)
Post-CBB feedback questions		
*How would you rate your experience taking this test?, n(%)*		
Poor	NA	489 (4.2%)
Fair	NA	2647 (22.9%)
Good	NA	5179 (44.8%)
Very Good	NA	2570 (22.2%)
Excellent	NA	617 (5.3%)
Response missing	NA	51 (0.4%)
*Were the instructions clear?, n(%)*		
Not Very Clear	NA	330 (2.9%)
Somewhat Clear	NA	2508 (21.7%)
Very Clear	NA	8622 (74.6%)
Response missing	NA	93 (0.8%)
*Do you think it would have been helpful for someone to explain this test to you and answer your questions before starting?, n(%)*		
Not Useful	NA	4497 (38.9%)
Not Very Useful	NA	3555 (30.8%)
Somewhat Useful	NA	2734 (23.7%)
Very Useful	NA	723 (6.3%)
Response missing	NA	44 (0.4%)
CBB Version, n(%)		
Flash	NA	5108 (44.2%)
HTML5	NA	6445 (55.8%)

Note. CBB: Cogstate Brief Battery

### Post-CBB feedback questions

The sample for this analysis was the total number of BHR participants aged 55+ who completed the post-CBB feedback questions the first time they took the CBB assessment (N=11,553). Overall, 44.8% (n=5179) rated their experience of taking CBB as “good”, 74.6% (n=8622) rated the instructions as “very clear”, and 30% (n=3457) rated additional human support with taking the CBB test as “somewhat useful or very useful”. See Table 1 for more information.

### Associations between sociodemographic variables and CBB feedback questions

Table 2 shows the results of ordinal logistic models to assess associations between sociodemographic variables and the ordinal post-CBB feedback questions.

**Table 2:** Estimated odds ratios and 95% confidence intervals from ordinal logistic regression models that assessed associations between participant characteristics and ordinal feedback responses

	**Sample size**	**OR (95%CI)**	**Type 3 p-value#**
How would you rate your experience taking this test?	N=11305		
Age (years/10)		0.78 (0.74–0.82)	
Levels of educational attainment			.0056
Post-secondary		1.0(reference)	
≤Secondary education		0.77(0.66–0.91)	
Postgraduate education		0.96(0.90–1.03)	
Gender			
Male		1.0(reference)	
Female		0.79 (0.74–0.86)	
Ethnocultural identity			<.0001
Non-Latino White		1.0(reference)	
Latino		0.74 (0.65–0.86)	
Non-Latino Asian		0.80 (0.61–1.04)	
Non-Latino Black		0.81 (0.61–1.08)	
Other non-Latino		0.63 (0.52–0.77)	
CBB version HTML5		1.0(reference)	
CBB version Flash		0.81 (0.76–0.87)	
Were the instructions clear?	N=11263		
Age (years/10)		0.72 (0.68–0.77)	
Levels of educational attainment			
Post-secondary		1.0(reference)	.58
≤Secondary education		0.91(0.75–1.11)	
Postgraduate education		1.02(0.93–1.11)	
Gender			
Male		1.0(reference)	
Female		0.96 (0.88–1.06)	
Ethnocultural identity			
Non-Latino White		1.0(reference)	<.0001
Latino		0.55 (0.47–0.65)	
Non-Latino Asian		0.65 (0.48–0.89)	
Non-Latino Black		0.69 (0.49–0.97)	
Other non-Latino		0.77 (0.61–0.98)	
CBB version HTML5		1.0(reference)	
CBB version Flash		0.65 (0.60–0.71)	
Do you think it would have been helpful for someone to explain this test to you and answer your questions before starting?	N=11312		
Age (years/10)		1.28 (1.22–1.35)	
Levels of educational attainment			.0017
Post-secondary		1.0(reference)	
≤Secondary education		1.17(1.00–1.38)	
Postgraduate education		0.91(0.85–0.98)	
Gender			
Male		1.0(reference)	
Female		0.92 (0.85–0.99)	
Ethnocultural identity			<.0001
Non-Latino White		1.0(reference)	
Latino		2.12 (1.85–2.44)	
Non-Latino Asian		1.96 (1.51–2.54)	
Non-Latino Black		1.95 (1.47–2.57)	
Other Non-Latino		1.22(1.01–1.49)	
CBB version HTML5		1.0(reference)	
CBB version Flash		1.26 (1.17–1.36)	

Note. OR: odds ratio; CI: confidence interval; CBB: Cogstate Brief Battery; # the type 3 p-value is a p-value for the composite null hypothesis that all levels of a categorical predictor have the same effect on the outcome as the reference category does.

Age was associated with the three post-CBB feedback questions. The associations were of modest strength. Specifically, 10 years increase in age was associated with decreased odds of rating the CBB test taking experience as excellent (OR=0.77, 95% CI:0.74–0.82) and rating the CBB instructions as useful (OR=0.72, 95% CI:0.76–0.77), as well as increased odds of rating additional human support as useful (OR=1.28, 95% CI:1.22–1.35).

Compared to participants who reported at least some level of post-secondary education, those with secondary education or less reported a significantly poorer CBB experience (OR=0.77, 95% CI:0.66–0.91). Those who reported a postgraduate education rated additional human support with taking CBB as less useful (OR=0.91, 95% CI:0.85–0.98) and those who reported secondary education or less rated additional human support with taking CBB as more useful (OR=1.17, 95% CI:1.00–1.38) compared to participants with at least some level of post-secondary, but not a postgraduate education.

Compared to registry participants who identified as non-Latino White, Latino participants rated their experiences of taking the assessment as significantly poorer (OR=0.74, 95% CI:0.65, 0.86), experienced the instructions as less clear (OR=0.55, 95% CI:0.47–0.65), and rated additional human support as more useful (OR=2.12, 95% CI:1.85–2.44). Non-Latino Asian and non-Latino Black participants also experienced the instructions as less clear (Asian: OR=0.65, 95% CI:0.48–0.89; Black: OR=0.69, 95% CI:0.49–0.97) and rated additional human support as more useful compared to non-Latino White participants (Asian: OR=1.96, 95% CI:0.1.51–2.54); Black: OR=1.95, 95% CI:1.47–2.57).

Self-identifying as female, compared to male, was associated with a poorer CBB test taking experience (OR=0.79, 95% CI:0.74–0.86) and rating additional human support as less useful (OR=0.91, 95% CI:0.85–0.99).

## Discussion

The major findings were that age, level of education, ethnocultural identity, and gender influenced how BHR participants experienced taking an unsupervised cognitive assessment for the first time. Specifically, increasing age, secondary education or less, and self-identifying as Latino, and female gender, were associated with a poorer CBB assessment taking experience; increasing age and self-identifying as non-White were associated with experiencing the test instructions as less clear; and higher age, and self-identifying as non-White and reporting secondary education or less were associated with rating additional human support with the test as more useful. The identified associations were of modest strength. These findings support the hypotheses that sociodemographic factors affect adults experience of unsupervised online cognitive assessment. These data therefore provide a foundation for strategies to improve methods for delivery and assessments of cognitive tests designed for remote and unsupervised application that could ultimately contribute to increased completion and retention by adults from communities that have typically been historically under-included in aging and dementia research.

The first major finding was that age was associated with CBB feedback responses.

Specifically, with increasing age, participants rated their CBB assessment experience as worse, rated CBB assessment instructions as less clear, and rated additional human support with the CBB assessment as more useful. The identified associations were of modest strength. Clarity of instructions is important to consider as it might affect the validity of the assessment results. Similar to our results, a previous study of the feasibility and acceptability of the CBB for remote use identified that the amount of time to read the instructions of one of the four tests increased with increasing age (9). Furthermore, another study found that CBB’s acceptability and usability was greatest in young- to middle-aged participants and that practice on the CBB prior to the assessment may have been beneficial for older participants, which the BHR CBB offers (20). It is also important to keep in mind that CBB was originally not developed as an unsupervised assessment which might explain some of the feedback in BHR. The CBB has been found to provide valid and feasible results across different settings (including unsupervised) (15, 23, 24), but in older adults location had an important impact on CBB performance (26). Despite increasing technology and internet use among older adults, studies of older adults’ technology adoption, also referred to as “gerontechnology adoption”, highlight the need for technologies to be designed with consideration for older adults’ needs and preferences in mind and have identified multiple barriers to adoption (27, 28). Adoption barriers of older adults particularly relevant to online cognitive assessments include incompatibility with older adults’ capabilities in terms of vision, hearing, and touch; less familiarity, experience, and confidence with the internet and cognitive assessments. A content analysis of participant feedback about taking the Montreal Cognitive Assessment via supervised Internet videoconferencing also revealed concerns about the effect of familiarity and accessibility of older adults with computers on the assessment experience (29). Similar to the above findings, an evaluation of a home-based dementia-related assessment trial found that participants requested more human contact (30). Future work could explore novel and scalable ways to offer more human contact in online remote settings (e.g., virtual live support, video assessment instructions) and identify additional avenues to improve the test taking experience and instructions for participants of varying ages.

The second major finding from this study was the level of education was associated with CBB feedback responses. The association was of modest strength. Specifically, compared to participants who had some level of post-secondary education but no post-graduate education, those with secondary education or less reported a poorer cognitive assessment taking experience and rated additional human support with taking CBB as more useful. Those with a postgraduate education rated additional human support with taking CBB as less useful. Contributing factors could be that many cognitive assessments are developed and tested in highly educated communities with often high levels of familiarity and use of technology and the internet compared to participants who received fewer years of education (31). However, cognitive assessments have also been shown to be appropriate in diverse communities when individuals are provided with the sufficient opportunities to practice (19, 20). This result might partly explain the results from a previous analysis which found that higher educational attainment was associated with higher cross sectional and longitudinal CBB completion in BHR (16). More work is needed to understand how the assessment experience could be improved for those with secondary education or less.

The third major finding from the current study was that compared to BHR participants identifying as non-Latino White, participants identifying as Latino, non-Latino Black, non-Latino Asian, and non-Latino Other Race experienced the test instructions as less clear and rated additional human support with instructions as more useful, which is consistent with previous research (30). In addition, participants who identified as Latino, also had a poorer test taking experience compared to non-Latino White participants. The identified associations with ethnocultural identity and CBB feedback responses were of modest strength. These findings might offer a partial explanation as to why the BHR and other AD research studies have so far failed to sufficiently engage non-White older participants to complete and return to complete online cognitive assessments (16). Further analysis is needed to investigate whether there is in fact an association between the completion of CBB and feedback about instructions. One possible explanation for our findings is that cognitive assessments are often developed and tested (including instructions) in non-Latino White populations (e.g., affected by cultural biases) (7, 8) and/or in in-clinic setting and later adapted. The CBB is considered by its developers to be a culture-free card test which has also been validated in Aboriginal communities in Australia (20), also a community often excluded from research. Even though the computerized version was validated, the Aboriginal participants received in-person support, which is different from the BHR setting. In addition, our sample is focused on ethnocultural communities prominent in the United States, so further validation would be necessaries for these communities. Further, the digital divide and limited assessment opportunities among ethnocultural communities could contribute to these findings (31). In addition, any individual with lower levels of familiarity with technology and cognitive assessment might benefit from more support when taking an assessment. Overall, these findings highlight the need for remote unsupervised cognitive assessments to be designed, developed, or adapted to adequately facilitate online cognitive assessment in diverse ethnocultural communities. Future research needs to identify specific changes that will improve the usability for diverse ethnocultural populations.

Finally, our analysis revealed that BHR participants self-reporting female gender rated their cognitive assessment experience as worse. In BHR, female participants have previously been found to complete CBB at baseline less frequently compared to male participants (16). This may be partly due to the worse CBB experience reported by female participants in this study. Even though the gender differences related to technology use have been shown to be narrowing, possible contributing factors to the reported findings could be less favorable female attitudes towards technology use compared to the males and a remaining bias towards male gender with technology (32, 33). Unlike our older participants, participants with a secondary education or less, and non-White participants, female participants indicated less interest in having human support with the assessment. Further investigation is needed to understand how we can improve the test taking experience of female participants.

This analysis is limited by BHR’s overall design and the voluntary nature of the provided feedback. BHR requires access to internet and a computer, as well as high literacy. In addition, BHR has only recently become available in both English and Spanish, and only a subset of participants provided the optional feedback. Therefore, the analysis is subject to multiple selection biases. Like other studies, our sample of participants who reported feedback underrepresents participants who identify as Latino, non-Latino Asian, non-Latino Black, other non-White, and male, as well as participants with an education less than a Bachelor’s degree. This impacts the generalizability of our findings. In addition, the participants who provided feedback may not represent the characteristics of the overall ethnocultural, educational, and gender populations being studied. For this analysis we also combined several ethnocultural populations into one ‘Other non-Latino’ group due to sample size concerns, but this did not allow us to explore potential feedback difference within the combined groups. Furthermore, our feedback questionnaire did not ask the respondents to clarify how the instructions and design could be improved, which needs to be investigated in the future. Further, the feedback questions’ five-point scale and the scale anchor terms (e.g., “fair”), as well as other self-report measures (e.g., levels of educational attainment) could be regarded as culturally biased. BHR also does not collect information about participants’ capabilities (e.g., vision, hearing, touch) or language fluency, which could impact the participant experience. In addition, this was a cross-sectional analysis of the first time a BHR participant provided CBB feedback. Future analyses should investigate if the identified associations remain when looking at feedback responses over time. Additional analysis could also focus on analyses of individuals whose assessment performance was within normal limits or include assessment performance measures in the analyses. This would allow to determine how much of the assessment experiences are related to assessment performance. Future analysis could also look at the other online cognitive assessment used in BHR and investigate which features best facilitate ease and accuracy in assessment given different cognitive styles and diverse ethnocultural backgrounds. Lastly, any cognitive assessment and design and instruction improvements need to be developed and tested in collaboration with the communities for whom we have failed to create an ideal cognitive assessment environment. The BHR has recently established two Community Science Partnership boards, one which includes Latino community members (17) and one with Black community members, which could facilitate this process.

Taken together, our findings point to the importance of improving unsupervised online cognitive assessment design and instructions to better suit the needs of diverse communities. Specifically, there is a need to improve the test taking experience and clarity of instructions, and to incorporate innovative, scalable ways to offer more human support for online remote assessments. This is the case especially for older adults, female participants, those with a secondary education and less, as well as commonly under-included ethnocultural communities. The results gained from this analysis can guide efforts to increase instruction comprehension and completion of unsupervised online assessments in diverse populations, but more research is needed to enhance our knowledge about concrete improvements in the assessment design and scalable digital ways to increase human support. These efforts need to take place in collaboration with the communities for whom we have failed to create an ideal cognitive assessment environment.
